# Review on neuroimaging in pediatric-type diffuse low-grade gliomas

**DOI:** 10.3389/fped.2023.1149646

**Published:** 2023-10-18

**Authors:** Jing Chen, Xin Qi, Mengjie Zhang, Jing Zhang, Tong Han, Chunxiang Wang, Chunquan Cai

**Affiliations:** ^1^ Department of Medical Imaging, Tianjin Children's Hospital (Tianjin University Children's Hospital), Tianjin, China; ^2^ Department of Magnetic Resonance, Lanzhou University Second Hospital, Lanzhou, China; ^3^ Department of Radiology, Tianjin Huanhu Hospital, Tianjin, China; ^4^ Tianjin Pediatric Research Institute, Tianjin Children's Hospital (Tianjin University Children's Hospital), Tianjin, China; ^5^ Tianjin Key Laboratory of Birth Defects for Prevention and Treatment, Tianjin, China

**Keywords:** brain tumor, gliomas, pediatric-type diffuse low-grade gliomas, 2021 WHO classification, neuroimaging

## Abstract

The fifth edition of the World Health Organization Classification of Tumors of the Central Nervous System (WHO CNS5) has identified a new classification system for tumors of the brain and spinal cord, highlighting the pivotal role of molecular diagnosis in accurately categorizing neoplasms. In addition to previous classifications, one of the key distinctions lies in categorizing pediatric-type diffuse low-grade gliomas (pDLGGs) and pediatric-type diffuse high-grade gliomas (pDHGGs) as distinct tumor types. Although similar in histology and morphology, pediatric diffuse gliomas are completely different from the adult type with respect to the molecular genetic characteristics, prognosis, and treatment strategies. pDLGG includes four tumor types, namely, diffuse astrocytoma, MYB- or MYBL1-altered; angiocentric glioma; polymorphous low-grade neuroepithelial tumor of the young (PLNTY); and diffuse low-grade glioma, MAPK pathway-altered, three types of which are newly recognized tumor types. Herein, we review the clinical characteristics, histopathological and molecular genetic characteristics, neuroimaging features, and prognosis of pDLGG and summarize the neuroimaging key points in diagnosing different tumor types. This review aims to evaluate and update the relevant pDLGG features and advances in neuroimaging that may assist in differential diagnosis, surgery planning, and prognostic determination of these tumor types and provide accurate diagnostic information for clinical colleagues.

## 1. Introduction

The 2016 World Health Organization (WHO) central nervous system (CNS) classification integrated molecular and histologic/phenotypic information for the first time (1). However, owing to advances in molecular diagnostics, the 2016 WHO CNS classification has some limitations in describing pediatric gliomas. The 2021 WHO CNS tumor classification includes molecular features for CNS tumors, adding new categories (2). The WHO CNS5 has classified diffuse gliomas into adult and pediatric types. Although pediatric diffuse glioma is similar to the adult type in histological morphology, its genetic characteristics and prognosis are molecularly distinct (3). Pediatric-type diffuse low-grade gliomas (pDLGGs) differ from adult forms with respect to molecular characteristics, biological behavior, clinical course, and prognosis. In the WHO CNS5, pDLGG is classified into four distinct tumor types, namely, (1) diffuse astrocytoma, MYB- or MYBL1-altered; (2) angiocentric glioma (AG); (3) polymorphous low-grade neuroepithelial tumor of the young (PLNTY); and (4) diffuse low-grade glioma, MAPK pathway-altered (Table 1). All four pDLGG tumor types are characterized by diffuse growth in the brain with overlapping histological features and poor specificity between them. Molecular analysis can be helpful in the characterization of lesions.

**Table 1:** WHO CNS5 classification of pediatric-type diffuse low-grade gliomas.

Subcategory	WHO grade	Genes altered	Molecular characteristics
Diffuse astrocytoma, MYB- or MYBL1-altered	1	MYB, MYBL1	MYB or MYBL1 rearrangements
Angiocentric glioma	1	MYB	MYB-QKI fusion
Polymorphous low-grade neuroepithelial tumor of young (PLNTY)	1	BRAF, FGFR family	FGFR2-CTNNA3 fusion
Diffuse low-grade glioma, MAPK pathway-altered	Not assigned	FGFR1, BRAF	TKD duplication, FGFR1 mutation, FGFR1 fusion

Low-grade gliomas are the most commonly encountered pediatric brain tumors. Although low-grade gliomas are a common type of CNS tumor in the pediatric age group, diffuse low-grade gliomas with infiltrative margins are relatively rare, with an incidence rate of only 8% (4). PDLGGs have a high incidence of BRAF p.V600E mutation, FGFR alteration, and/or MYB or MYBL1 rearrangement (5, 6).

According to the previous WHO classification criteria of CNS tumors, the prognosis of these tumors varies greatly. Here, we review the imaging and pathological features, clinical manifestations, treatment, and prognosis of pDLGG, aiming to enhance our comprehension of the disease and provide an initial interpretation from the perspective of clinical diagnosis and treatment. Table 2 shows the neuroimaging features of pDLGG (7–12).

**Table 2:** Neuroimaging features of pDLGG.

	Location	Boundary	Enhancement	Edema	Typical features
Diffuse astrocytoma, MYB- or MYBL1-altered	Temporal lobe	Clear	None	Mild	Iso- to hypointense on T_1_WI and mixed-signal or hyperintense on T_2_WI/FLAIR
Angiocentric glioma (AG)	Temporal lobe, tends to be superficially located	Well circumscribed	No or slightly patchy	Mild	Intratumoral T_1_WI high-intensity area, stalk-like sign, and atrophy of the adjacent brain parenchyma
Polymorphous low-grade neuroepithelial tumor of young (PLNTY)	Temporal lobe, more common on the right	Ill-defined	Slight or no enhancement	Uncertainty	Granular calcification, “salt and pepper sign” on T_2_WI
Diffuse low-grade glioma, MAPK pathway-altered	Cerebral cortical region	Unclear	Obvious and heterogeneous	Mild	Calcification is more common
	Diencephalon	Unclear	Obvious and homogeneous	Mild	Mostly solid and lobular

## 2. Characteristics of pDLGGs of different tumor types

### 2.1. Diffuse astrocytoma, MYB- or MYBL1-altered

Diffuse astrocytoma with alterations of MYB/MYBL1 is a diffuse, infiltrating tumor composed of astrocytic cells. Its histological appearance is not distinguishable from that of astrocytic tumors. The tumor belongs to the family of MYB/MYBL1-altered gliomas and is an *IDH*-wild type. It exhibits low-grade histopathologic features and represents a distinct group of tumors with indolent behavior and a favorable prognosis (9). The tumor cell proliferation index is low and usually classified as WHO grade 1 (13).

#### 2.1.1. Genetic and histopathological alterations

*MYB* is a proto-oncogene that plays an essential role in controlling the proliferation and differentiation of hematopoietic and other progenitor cells and acts as a proto-oncogene in leukemia and other solid tumors (14). *MYBL1* is closely associated with MYB and has a similar function (15). Alterations of these genes have been associated with various hematologic diseases and solid malignancies. The alterations include MYB or MYBL1 rearrangements and MYB-QKI fusion (5). The MYB/MYBL1 alterations are associated with diffuse astrocytoma, while the MYB-QKI fusion is highly relevant to AG. Diffuse astrocytoma, MYB- or MYBL1-altered, is a diffuse infiltrative neoplasm composed of astrocytoid cells that exhibit histological morphology indistinguishable from conventional astrocytomas. The tumor exhibits invasiveness and primarily affects the cerebral hemispheres (16).

#### 2.1.2. Clinical and prognostic significance

Patients were primarily young children (median age: 5 years; range 0–26 years), and no gender predilection was observed (17). The tumors most commonly involved the cerebral cortex, followed by cerebral white matter and/or deep gray nuclei (18). Most patients with diffuse MYB/MYBL1-altered astrocytoma have a history of epileptic seizures since childhood (13). Furthermore, symptoms such as movement disorders and behavioral changes occur in both encephalopathy and glioneuronal tumors (19).

The prognosis of diffuse astrocytoma with alterations of *MYB/MYBL1* is generally excellent. Chiang et al. (17) found that the 10-year progression-free survival and overall survival rates were 89.6% and 95.2%, respectively. The conservative approach is recommended for inert IDH-wild-type diffuse astrocytoma owing to its more favorable prognosis and progression-free survival (9). Yang et al. (20) found that children with *MYB* amplification have a better prognosis and longer progression-free survival than those without *MYB* amplification.

#### 2.1.3. Imaging features

**Figure 1:**
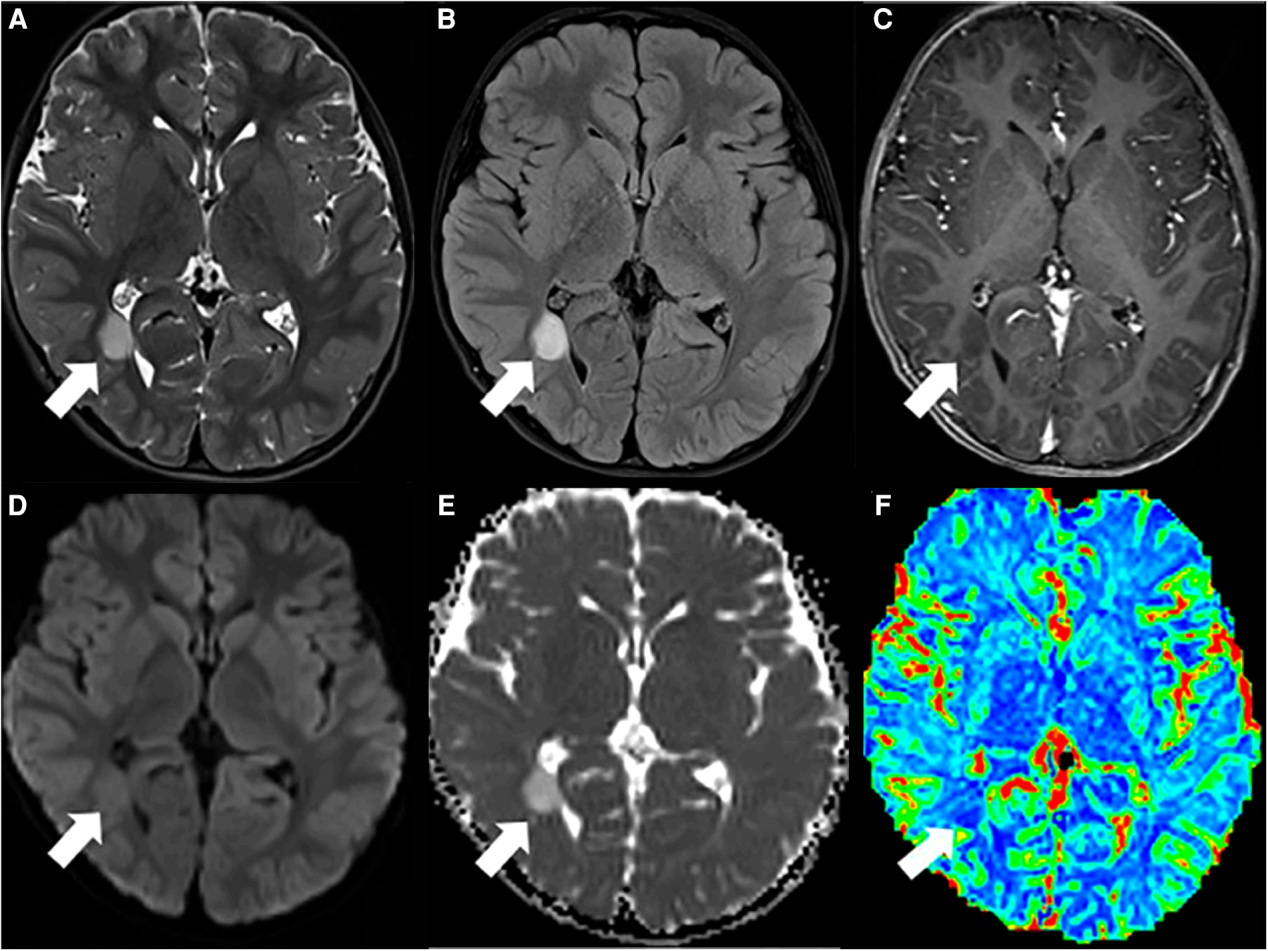
Pediatric-type diffuse low-grade glioma. (**A**) T_2_WI and (**B**) FLAIR hyperintense lesion in the right temporo-occipital (arrow). (**C**) There is no enhancement on the T_1_WI postcontrast image (arrow). (**D**) Facilitated diffusion seen on diffusion-weighted imaging (DWI) (arrow) and (**E**) ADC map (arrow). (**F**) Hypoperfusion is seen on the rCBV map (arrow). This figure was originally posted by Kalelioglu et al. in the *Neuroradiology Journal* in September 2022 (9).

The imaging findings of this tumor type are similar to those of adult diffuse low-grade glioma, with invasive growth mainly involving the cerebral hemispheres. The brain stem and diencephalon are less commonly involved (18). The tumors are most commonly found in the temporal lobe, followed by the frontal and occipital lobes. They commonly involve the cortical and subcortical regions of the cerebral cortex (21). The tumors displayed a mass effect and mild peritumoral edema. The lesion showed an iso- to hypointense signal on T_1_-weighted imaging (WI) and mixed-signal or hyperintense signal on T_2_WI/fluid-attenuated inversion recovery (FLAIR) (Figure 1) (9, 17), without evidence of restricted diffusion. Contrast enhancement was never observed. Magnetic resonance spectroscopy (MRS) shows increased choline and decreased N-acetyl-aspartate (18, 22).

#### 2.1.4. Differential diagnosis

Diffuse astrocytoma should be distinguished from oligodendroglioma and diffuse hemispheric glioma. Oligodendroglioma is mostly located in the frontal lobe and typically shows gyriform calcification (23). Diffuse hemispheric glioma belongs to pediatric-type diffuse high-grade gliomas (PDHGGs), with poor prognoses. The tumors most commonly involve the frontal lobe and parietal lobe, particularly the profound nuclei. The signal intensity on MRI images is intricately modulated by the coexistence of cystic, hemorrhagic, and necrotic constituents. They exhibit invasive growth patterns with indistinct margins and enhancement, with restricted diffusion (24, 25).

### 2.2. Angiocentric glioma

AG is a rare cortical/juxtacortical epilepsy-related low-grade glioma (WHO grade 1) that occurs in children and young adults (7). It is defined by characteristic *MYB-QKI* gene fusion and shows angiocentric patterns upon histopathological examination (14).

#### 2.2.1. Genetic and histopathological alterations

*MYB* is a proto-oncogene, and *MYB* proteins are transcription factors characterized by a highly conserved DNA-binding base sequence. *MYB-QKI* fusion is relevant to AG (8). MYB-QKI fusion is found in 87% of AGs and 41% of pDLGGs (18). The functional consequence of this fusion is the loss of the tumor suppressor function of QKI combined with the activation of MYB (21, 26). The histopathology of AG is characterized by “monomorphous bipolar fusiform cells” and “an angiocentric growth pattern,” which means that the tumor consists of monomorphic cells that align longitudinally around blood vessels, and the neurons form pseudo-rosettes (27). The tumor demonstrates indistinct boundaries and closely associates with the cerebral tissue. The tumor cells exhibited an elongated and bipolar morphology under microscopic examination, arranged in a radial pattern around the affected blood vessels and oriented vertically or parallel to the vascular endothelial cells (8).

#### 2.2.2. Clinical and prognostic significance

Patients <20 years of age are the most affected, with no sex differences (8). The vast majority of patients have a long history of intractable seizures. The disease course is often accompanied by seizures owing to AGs that tend to be located superficially. Other clinical symptoms include facial weakness, double vision, irregular gait, and headache with vomiting (7).

AG is a slow-growing, stable tumor with a good prognosis after surgical resection, and the incidence of tumor recurrence after complete resection was low (7). Seizures/epilepsies improve when complete resection is performed (28).

#### 2.2.3. Imaging features

**Figure 2:**
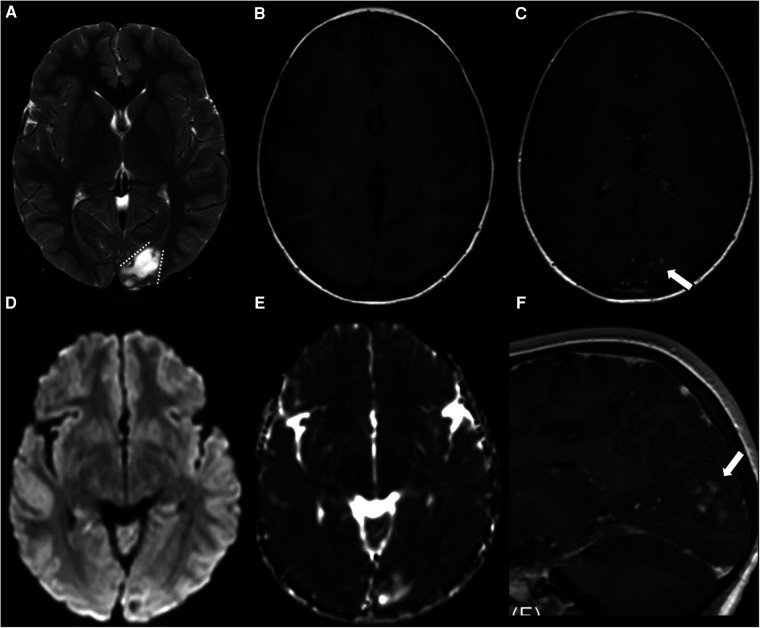
Supratentorial angiocentric glioma in a 10-year-old boy. The tumor shows high intensity on T_2_WI (**A**) and low intensity on T_1_WI (**B**). The stalk-like sign is observed without evidence of atrophy in the surrounding brain parenchyma (**A**, dotted lines). Diffusion restriction is not observed (**D,E**). Nodular enhancement is observed in the postcontrast sagittal T_1_WI (**C,F**, thick arrows). This figure was originally posted by Kurokawa et al. in the *Journal of Neuroimaging* in 2022 (7).

AG is typically localized in the supratentorial cortex and subcortical white matter, predominantly affecting a single lobe, with the temporal lobe being the most commonly involved, followed by the frontal lobe, parietal lobe, brain stem, and thalamus, mostly involving the hippocampus (29, 30). The neuroimaging features of AG vary, showing low density, high density, or mixed density on computed tomography (CT), and calcification is generally rare. In some instances, a hyperdense lesion was observed on a CT scan, potentially indicating the presence of hemorrhage and deposition of hemosiderin (29). AG has a characteristic MRI appearance comprising an intratumoral T_1_WI high-intensity area, stalk-like sign, and atrophy of the adjacent brain parenchyma (Figure 2) (7). The stalk-like sign indicates that the tumor is located along the vessels extending from near the brain surface toward the ventricle. Solid ingredients showed isointense, hypointense, or hyperintense signals on T_1_WI and T_2_WI/FLAIR. Cystoid components are common and showed a hypointense signal on T_1_WI and a hyperintense signal on T_2_WI/FLAIR. Contrast enhancement was observed in more than 25% of patients despite the general consensus that AGs are considered to have no contrast enhancement. Typically, diffusion restriction in AGs is also atypical. On MRS, elevated levels of creatine and choline and decreased levels of NAA have been described, and lactate peaks were observed (8, 18, 31, 32). It is a well-circumscribed tumor in that peripheral edema and mass effect are usually absent (33). In addition, some studies have reported the coexisting nature of adjacent focal cortical dysplasia (FCD) (7).

#### 2.2.4. Differential diagnosis

The differential diagnosis includes ganglioglioma, pleomorphic xanthoastrocytoma (PXA), and dysembryoplastic neuroepithelial tumor (DNET) (32). Gangliogliomas typically consist of cystic components and calcification, with approximately 50% demonstrating contrast enhancement. However, AGs generally lack such enhancement (19). On a CT scan, PXA shows hemorrhage more commonly, but calcification is rare (34). There is typically adjacent cortical thickening and fewer calcifications in DNET (10).

### 2.3. Polymorphous low-grade neuroepithelial tumor of the young

PLNTY is an epileptogenic tumor first described by Huse et al. in 2017, described as an epileptogenic histopathological tumor type with specific histological, immunohistochemical, and molecular profile combining an oligodendroglioma-like component, diffuse CD34 expression, and genetic alterations in the MAP kinase pathway (35).

#### 2.3.1. Genetic and histopathological alterations

An immunohistochemical examination reveals obvious differentiation of glial cells and intense positivity for CD34 and commonly shows abnormal MAPK pathway alterations. The expression of CD34 may be strong and diffuse or show focal distribution. Strong positivity for GFAP and OLIG2 could also be commonly seen. It is generally negative for IDH1, R132H, EMA, NeuN, and neuroendocrine markers, and the MIB-1 tag index is often low (<1%–2%). Molecular profiling of PLNTY shows that it carries a distinct DNA methylation signature, including a potential epigenetic subgroup defined by FGFR2 fusions. Molecular analysis of PLNTY includes genetic abnormalities involving either BRAF V600E mutation (≈40%) or FGFR 2/3 fusion (≈50%); common fusion genes include *FGFR2-KIAA1598*, *FGFR2-CTNNA3*, and *FGFR-TACC3* (15).

Macroscopical examination reveals a generally well-circumscribed solid–cystic tumor with calcified and cystic components at the periphery that are unencapsulated. Histological examination reveals that characteristic manifestations of PLNTY are oligodendrocyte tumor-like components associated with other components, including fibrillary, fusiform, spindled, or pleomorphic astrocytic cells. Vague perivascular pseudorosettes are occasionally described, and the adjacent cortex may show evidence of FCD (36). Variable degrees of calcification are frequently observed in the tumor, which usually appears coarse and confluent.

#### 2.3.2. Clinical and prognostic significance

PLNTY often occurs in children and young adults and shows slight female predominance (37). The median age of presentation is approximately 16 years (range: 4–57 years). The most prevalent clinical manifestation is epilepsy, also referred to as long-term epilepsy-associated brain tumors, which includes pilocytic astrocytoma, ganglioglioma, PXA, DNET, AG, pediatric oligodendroglioma, and other related conditions (23). Other clinical manifestations include headache, dizziness, and visual impairment mainly related to the tumor location.

Because of the very low proliferation rate, the WHO criteria currently classified PLNTY as grade 1. It often tends to exhibit a benign biological behavior and clinical course, most of which can be well controlled by gross total resection (36). However, a case report of malignant transformation of PLNTYs demonstrated *FGFR3* (exon 18) and *TACC3* (exon 11) fusion associated with additional somatic alterations in *TP53*, *ATRX*, *PTEN*, *TEK*, and *RB1* consistent with more aggressive biology, typically seen in high-grade glioma (38). Therefore, although most cases are benign, long-term follow-up of PLNTY is warranted. Tateishi et al. (39) revealed that the activation of MAPK signaling and subsequent c-Myc induction, driven by the BRAF V600E mutation, elicit specific metabolic alterations in PLNTY cells. The superior potential of targeted drugs in unresectable regions is suggested.

#### 2.3.3. Imaging features

**Figure 3:**
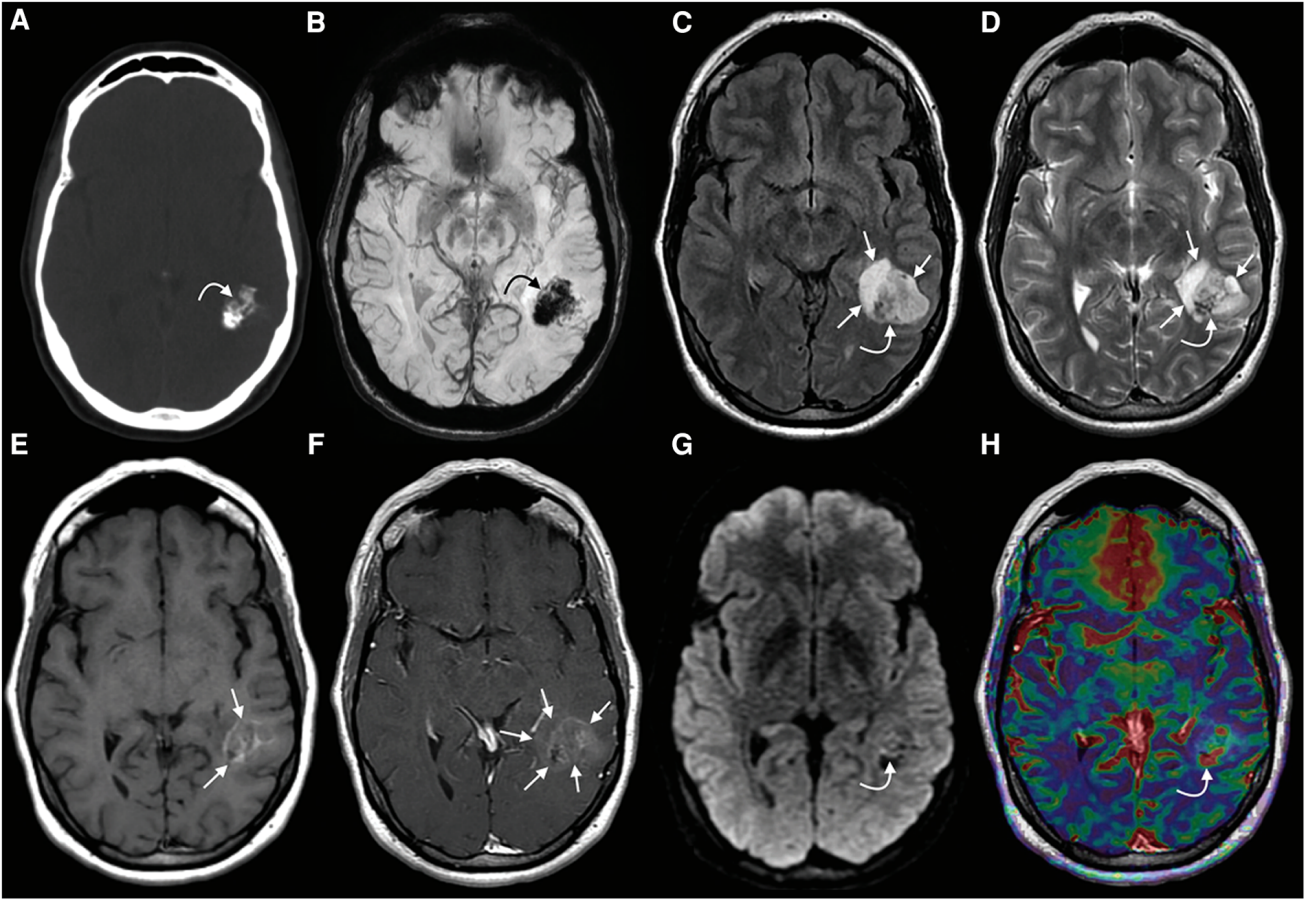
PLNTY. CT (**A**) image showing a mass within the left temporal lobe with dense intralesional calcifications, with a blooming signal on the corresponding SWI (**B**) (curved arrows in **A** and **B**). A heterogeneous signal is noted on both FLAIR (**C**) and T_2_WI (**D**) (curved arrows on **C** and **D**). A faint T1-hyperintense signal is noted in the central components of the tumor (straight arrows, **E**), while a greater extent of the mass demonstrates mild enhancement (straight arrows, **F**). A few tiny foci of mildly restricts diffusion were observed centrally (curved arrow, **G**). Some of the solid-appearing components demonstrates elevated relative CBV (curved arrow, **H**). This figure was originally posted by Benson et al. in *AJNR* (*American Journal of Neuroradiology*) in April 2020 (41).

**Figure 4:**
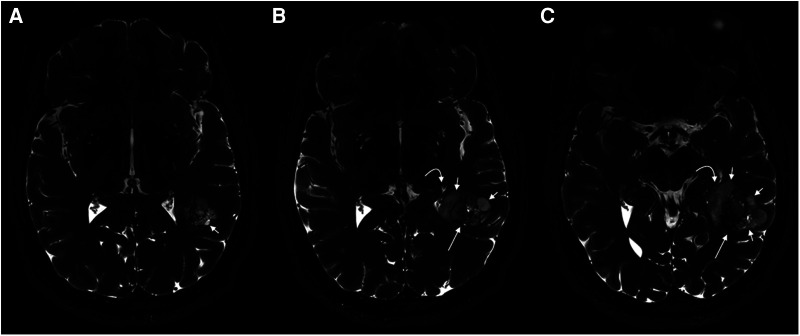
PLNTY. 7T MR imaging demonstrates internal characteristics of the mass on T_2_WI. From left to right (**A–C**), heterogeneous solid material is seen centrally (long straight arrows), while cystic components are located peripherally (short straight arrows). A mild associated mass effect is seen. This figure was originally posted by Benson et al. in *AJNR* (*American Journal of Neuroradiology*) in April 2020 (41).

The majority of PLNTY arises in the cerebral hemispheres, with a predilection for the temporal lobe, followed by the occipital lobe, frontal lobe, and parietal lobe. These regions of the brain are commonly located in the cortical and subcortical areas with clearly circumscribed margins. Approximately 80% of tumors are found in the temporal lobe, predominantly on the right side, but rarely in the ventricles (40). PLNTY has typical imaging features (Figures 3, 4) (41). On the CT scan, granular calcification in the tumor is one of the key radiological features. On magnetic resonance imaging (MRI), PLNTY shows the most commonly heterogeneous iso- to hypointense signal on T_1_WI and hyperintense signal on T_2_WI/FLAIR sequences with local slight or no enhancement after contrast enhancement (23). A cystic component can be present in the majority of PLNTY. No restricted diffusion is noted on the diffusion-weighted imaging sequence. Perfusion-weighted imaging shows increased focal cerebral blood volume. The “salt and pepper sign” on T_2_WI may be a distinctive manifestation of PLNTY, potentially attributed to calcification within the grit (42). In addition, cortical dysplasia is frequently observed in conjunction with low-grade tumors (43).

#### 2.3.4. Differential diagnosis

PLNTY must be distinguished from ganglioblastoma, oligodendroglioma, and DNET, all of which are common in the temporal lobe (37). Ganglioblastoma is another epilepsy-relevant tumor, and it always appears as an isointense signal to the gray matter on T_1_WI (19, 44). Calcifications and cystic components are more common in PLNTYs than ganglioblastoma. There are usually more intratumoral septations and fewer calcifications in DNET (10). The characteristic gyriform calcification helps distinguish oligodendroglioma from PLNTY (23).

### 2.4. Diffuse low-grade glioma, MAPK pathway-altered

Diffuse low-grade glioma, MAPK pathway-altered is also a newly defined tumor type in the 2021 WHO CNS5, which requires a combination of histological evaluation and molecular characterization to diagnose. However, different from the other three types of pDLGG defined as WHO grade 1, the specific WHO grade classification of the diffuse low-grade glioma, MAPK pathway-altered is not assigned, which may be related to the small number of cases and lack of sufficient clinical course data.

#### 2.4.1. Genetic and histopathological alterations

Histological examination reveals minimal and atypical glial proliferation and diffuse astrocytic or oligodendroglial morphology, similar to other WHO grade 2 diffuse gliomas. In immunohistochemical examination, compared with angiocentric gliomas, diffuse low-grade glioma, MAPK pathway-altered lacks the characteristic growth pattern around the blood duct center, is EMA spot-positive, and shows MYB variation. In contrast to PLNTY, diffuse low-grade glioma, MAPK pathway-altered does not have a strong diffuse positive expression of CD34 (45). BRAF V600E mutation and FGFR1 alteration are commonly found upon molecular analysis. Common molecular alterations of diffuse low-grade glioma, MAPK pathway-altered include TKD duplication, FGFR1 mutation, FGFR1 fusion, BRAF V600E mutation, BRAF fusion, or BRAF insertion mutation (5).

#### 2.4.2. Clinical and prognostic significance

MAPK pathway-altered is a rare tumor with a diffuse and invasive growth pattern, which mainly occurs in children, is commonly associated with epilepsy, and occasionally affects adults (26). Yang et al. (20) classified diffuse low-grade glioma, MAPK pathway-altered with BRAF V600E mutation into the intermediate risk group.

#### 2.4.3. Imaging features

**Figure 5:**
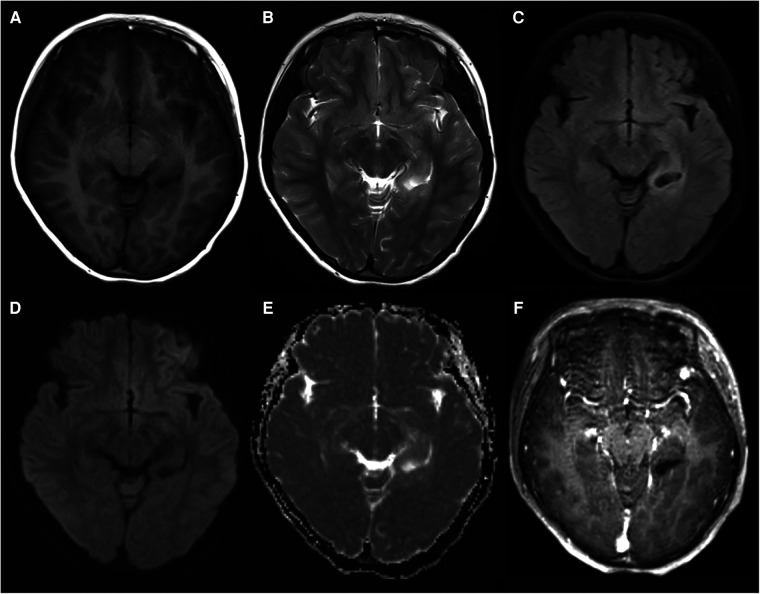
Pediatric-type diffuse low-grade glioma, MAPK pathway-altered in a 10-year-old girl. The tumor is located in the left hippocampus and shows hypointense on T_1_WI (**A**) and hyperintense on T_2_WI (**B**) and FLAIR (**C**). Diffusion is not restricted (**D,E**). No enhancement is observed (**F**).

The imaging features of diffuse low-grade glioma, MAPK pathway-altered are related to the site and histological examinations. For tumors located in the cerebral cortical region, calcification is more common; hypointensity on the T_1_WI sequence and hyperintensity on the T_2_WI sequence with obvious and heterogeneous enhancement after contrast enhancement are typically seen (12). In some cases, both cystic and solid components can be observed instead of calcification. Atypically, no obvious enhancement was observed (Figure 5). The tumors in the diencephalon predominantly exhibit solid and lobular characteristics, demonstrating obvious and homogeneous enhancement without any signs of necrosis, edema, and space-occupying effects (46).

#### 2.4.4. Differential diagnosis

The primary differential diagnosis includes ganglioblastoma, DNET, and PLNTY. Both ganglioblastoma and DNET have been previously mentioned. Granular calcification and the “salt and pepper sign” on T_2_WI are characteristic features of PLNTY. Furthermore, PLNTY exhibits minimal or negligible enhancement after contrast enhancement, while diffuse low-grade glioma, MAPK pathway-altered demonstrates conspicuous enhancement with contrast.

## 3. Conclusion

The revised tumor classification reflects the comprehensive understanding of CNS tumors by domain experts in light of current scientific knowledge. With the widespread implementation of novel detection techniques, an increasing number of novel molecular variants associated with tumors will be identified. Simultaneously, as the relevant clinical trials progress, our comprehension of the classification system for CNS tumors will be further enhanced.

MRI is the first choice and optimal imaging modality for evaluating pediatric CNS tumors. It accurately depicts various imaging features of the tumor, including size, margin, morphology, location, signal characteristics, mass effect, peritumoral edema, and enhancement features. CT is particularly sensitive in detecting subtle calcifications. In summary, we have highlighted the distinctive features of pDLGG to enhance the understanding of these tumor types and provide additional diagnostic information.
